# Childcare Center Characteristics Moderate the Effects of a Physical Activity Intervention

**DOI:** 10.3390/ijerph17010101

**Published:** 2019-12-22

**Authors:** Ruth P. Saunders, Marsha Dowda, Karin A. Pfeiffer, William H. Brown, Russell R. Pate

**Affiliations:** 1 Department of Health Promotion, Education, and Behavior, Public Health Research Center, University of South Carolina, 921 Assembly Street, Suite 212, Columbia, SC 29201, USA; 2 Department of Exercise Science, Public Health Research Center, University of South Carolina, 921 Assembly Street, Suite 212, Columbia, SC 29201, USA; mdowda@mailbox.sc.edu (M.D.); rpate@mailbox.sc.edu (R.R.P.); 3 Department of Kinesiology, Michigan State University, 308 West Circle Drive, 27R Intramural Rec Sports-Circle, East Lansing, MI 48824, USA; kap@msu.edu; 4 Educational Studies, Wardlaw College, University of South Carolina, 820 Main Street, Columbia, SC 29208, USA; bbrown@mailbox.sc.edu

**Keywords:** early childhood education and care, preschool, physical activity, intervention studies

## Abstract

Center-based early childhood education and care (ECEC) programs are well-positioned to create positive impacts on the health and development of large numbers of young children by promoting physical activity using evidence-based programs. Studies testing physical activity programs for young children should examine the circumstances under which programs are most effective by assessing the role of contextual factors on program outcomes. The purpose of this study was to examine the moderating effects of baseline ECEC center characteristics on the relationship between the Study of Health and Activity in Preschool Environments (SHAPES) intervention and moderate-to-vigorous physical activity (MVPA). MVPA was assessed via accelerometry; center characteristics, practices, and social and physical environments were assessed by director interview and observation; and center quality was assessed using the Early Childhood Environment Rating Scale-Revised Edition. Mixed-model analyses of covariance (ANCOVAs) examined intervention effects on MVPA during the school day; interactions between baseline center variables and group assignment (intervention vs. control) tested for moderation. Two center instructional practices, two social environment characteristics, and one physical environment characteristic at baseline moderated the effects of SHAPES on MVPA outcomes. Assessing baseline practices and center characteristics may aid efforts to match centers with interventions likely to increase physical activity as well as suggest additional intervention strategies to test.

## 1. Introduction

Physical activity is associated with better physical, social, and psychological health among young children [1,2]. Center-based early childhood education and care (ECEC) programs serve large numbers of young children [3,4] and are therefore well-positioned to create a positive impact on child health and development by providing opportunities for physical activity [5]. The Institute of Medicine (IOM), now the National Academy of Medicine, recommends that early childhood settings provide daily indoor and outdoor physical activity opportunities for at least 15 min per hour, and the Society of Behavioral Medicine recommends increasing physical activity during child care hours to 120 min per day [5]. However, many young children in these settings are insufficiently active [6,7,8,9].

ECEC settings are well-suited for using social ecological approaches to promote physical activity [10,11] because compared to cognitive factors, environmental factors have a greater influence on young children’s behavior [12]. Center policies, practices, and environmental characteristics are known to exert important influences on children’s physical activity levels [13,14,15,16,17]. Therefore, interventions in these settings ideally should be guided by multilevel approaches, including a focus on influential environmental components.

Social ecological approaches posit that behavior (the outcome variable) is influenced by factors (determinants or independent variables) at multiple levels (e.g., individual, social, organizational, physical, environmental) and that independent variables influence each other [10,11]. Intervention studies typically examine effects of the intervention on the outcome and determinants separately, without considering interactions between the intervention effects and determinant variables [18]. Full operationalization of a social ecological approach to assessing the impact of an intervention should address the question: “For whom and under what circumstances does the intervention work?” [19,20]. If a characteristic of the target audience (e.g., gender) or a feature of the setting (e.g., playground size) affects the direction or strength of the intervention, it is a moderator [18,19,20]. Moderating variables are tested by including an interaction term into the outcome analysis [18,19].

Few studies have investigated moderators and most of these examined the role of individual-level factors, such as gender [21], rather than that of environmental-level factors [8,12]. However, efforts to maximize the positive public health impact of promoting physical activity in ECEC settings also require understanding how the context influences the effects of policy, practice, and environmental change interventions (i.e., under what circumstances the intervention is effective). Understanding the influence of both individual and environmental factors will enable researchers to match intervention strategies to specific types of settings and to tailor interventions for specific populations.

The Study of Health and Activity in Preschool Environments (SHAPES) was an environmentally focused intervention guided by a social ecological approach to promote physical activity in center-based ECEC settings. As previously described [22,23], the goal of SHAPES was to increase physical activity in young children by providing training and support to modify teachers’ instructional practices in three key settings: indoors (“Move Inside”), outdoors (“Move Outside”), and preacademic lessons (“Move to Learn”) to provide 300 min of physical activity weekly. SHAPES also equipped teachers with the skills to modify the social and physical environments to promote physical activity. Each teacher determined the optimal combination of Move Inside, Move Outside, and Move to Learn that best fit her classroom and center resources and teachers were encouraged to overcome barriers such as limited space in creative ways. The resulting flexible, ecological physical activity intervention was effective in increasing objectively assessed moderate-to-vigorous physical activity (MVPA) in young children; children in the intervention centers engaged in significantly more MVPA than children in control centers (7.4 and 6.6 min per hour, respectively) [23].

SHAPES provided an opportunity to examine how baseline characteristics of early childhood education and childcare settings (center demographic, policy and practice, and social and physical environment variables) moderated the influence of the SHAPES intervention on the primary outcome, MVPA. The purpose of this study was to examine the moderating effects of baseline center practices and social and physical environment characteristics on the relationship between the center-based SHAPES intervention and MVPA. In this exploratory study we anticipated that the SHAPES intervention would have larger or smaller effects in childcare centers with varying baseline characteristics.

## 2. Materials and Methods

### 2.1. Design and Setting

As previously reported [23], the Study of Health and Activity in Preschool Environments (SHAPES) used a group randomized design with center as the unit of randomization and analysis. Childcare centers in the Columbia, SC area meeting eligibility requirements including a focus on developmental and pre-academic skills, adherence to state curriculum standards, and program length of ≥ 3 h/day and ≥ 180 instructional days per year were included in the study. Sixty-two public and private centers that met the eligibility criteria were identified, and a stratified random sample of 16 centers was invited to participate in the study. If a center declined to participate, another school from the same stratum was invited. The 16 childcare centers that agreed to participate were pair-matched by type (public or private), number of students enrolled, number of classrooms for 4-year-olds, and number of children per classroom. Centers from each pair were randomly assigned to either the control or intervention condition. Data were collected in two consecutive 4-year-old cohorts (waves) of students (2008–2009 and 2009–2010) from the 16 centers. Baseline measures were administered in the fall of the school year and follow-up measures in the spring.

### 2.2. Study Participants

Parents of all children enrolled in classrooms for 4-year-olds were invited to participate in the measurement protocol. The recruitment goal per center was 15 children; 378 children participated. Centers were randomized into control and intervention with eight centers in each group. The study was approved by the University of South Carolina’s Institutional Review Board (PRO#00004884).

### 2.3. Measures

Potential moderating variables reflected a multi-level social ecological framework and included baseline demographics, center policies and practices, organizational characteristics, and center physical and social environments. Physical activity was assessed via accelerometry at baseline and post-intervention.

*Demographics.* For each center, enrollment figures were used to determine child demographics for the classrooms with 4-year-olds, including percentage that was male and the percentage that was black. Participating parents reported their education and were categorized as having less than or greater than/equal to 2 years of college education or more. Centers were then dichotomized as having < or > 50% male students, < or > 70% black students, and < or > 50% parents with 2 years of college/tech school or more.

*Center Policies, Practices, and Characteristics*. At each wave of data collection, center directors/principals completed a structured interview about center-level policies and practices. The procedure for coding data was based on that used by Dowda and colleagues [16]. Directors were asked about teachers’ instructional practices, specifically the provision of daily free play (15–30 min, 31–45 min, 46–60 min, or 60+ min per day); centers were dichotomized as providing < or ≥ 46 min free play per day. Questions also assessed teacher-led structured physical activity days per month and minutes per session (< 20 min, 20–30 min, 30–45 min, 45 min+); centers were dichotomized as having this type of daily activity (no or yes) and < or > 30 min per session. Finally, staff participation in free play with children was assessed (rarely/sometimes vs. often/always join children).

Other policy and practice items included restricting active play for misbehavior (often/sometimes vs. never/reward for good), TV viewing (> or ≤ once per week), off-site trips (≤ 1 or > 1 trip per month), and teacher physical activity training (no or yes). One organizational characteristic of the centers, public vs. private, was also assessed.

*Center Physical and Social Environments*. Trained research staff used the SHAPES Inventory Assessment to measure classroom and playground size and get counts of fixed playground equipment, and used the SHAPES Process Observation form to obtain counts of portable equipment and children per classroom; both observational instruments, described previously, were developed for this study [16,22]. Classroom and playground measurements were divided into tertiles, and classroom size and playground size were dichotomized as the two lower tertiles vs. the higher tertile. Counts of fixed equipment and number of children per classroom were divided into quartiles and dichotomized as the lower three quartiles vs. the upper quartile. Data on the amount and quality of portable playground equipment were obtained from the director survey and dichotomized as little/some variety vs. good/lots of variety [16].

One item on the director survey assessed the physical activity social environment, specifically, visible support and messages promoting physical activity (none/few vs. display posters/books about physical activity). Overall center quality was assessed using the Early Childhood Environment Rating Scale-Revised Edition (ECERS-R) [24]. It was administered in one randomly selected classroom per center by a trained researcher. The ECERS-R provides an estimate of the level of center quality based on current understanding of best practices in early childhood education, and higher scores have been positively associated with young children’s MVPA [16].

The ECERS-R elements that best predict child educational outcomes are those that pertain to student–teacher interactions [25]. The social environment is also an important influence on physical activity in this setting [26]. Accordingly, a subset of 10 ECERS-R items was used to create a social environment score. Items were drawn from several domains and included greetings when children were arriving and departing, encouraging children to communicate, staff–child interactions, and interactions among children; centers that ranked in the upper 75% of these scores were compared to those in the lower quartile.

*Physical Activity*. Physical activity was measured over five days (Monday–Friday) using Actigraph GT1M and GT3x accelerometers, which were distributed by data collection staff. Data were collected and stored in 15-s intervals. Children wore the monitors on an elastic belt on their right hip. Parents were instructed to remove the monitor only during water-related activities, such as bathing, and when the children went to bed at night.

Data were reduced using activity intensity cut-points developed specifically for children 3–5 years; the moderate-to-vigorous cut-point was ≥ 420 counts/15 s [27]. Sixty minutes of consecutive zeros were considered as non-wear time [23]. For this study, only time in the center was used in the analyses. Minutes/hour of MVPA were calculated by using children’s wear time during the center day as the divisor. A day of observation was considered compliant if a child provided accelerometry data for ≥ 50 % of the school day. Children who were compliant with ≥ 3 days of accelerometer data were included in the analyses.

### 2.4. Statistical Analysis

Chi-square analyses were conducted to determine if there were statistical differences between the control and intervention groups for the proposed baseline moderators. The *p*-value for this analysis was < 0.05.

As with the primary analysis [23], mixed-model ANCOVAs were calculated to determine the effects of the intervention on MVPA during the school day. To test for moderation, a possible baseline moderating variable and an interaction of the moderator with the group (intervention vs. control) was added to the ANCOVA; *p*-values < 0.05 for the interactions between group and center characteristics were considered significant [19].

All models were adjusted for baseline MVPA and wave, and center was treated as a random variable which accounted for clustering. MVPA was skewed; therefore, both pre- and post-intervention MVPA were square root transformed. Missing values at follow-up were replaced for 30 children in the control group and 22 in the intervention group using multiple imputation as previously reported [23]. Untransformed least-square means for the interaction between intervention group and moderator were reported. Stratified analyses were conducted for variables with significant interactions and least-square means of MVPA were calculated.

## 3. Results

At the child level, (Table 1) the intervention and control groups had similar percentages based on sex, age, and body mass index (BMI). Parent education was higher in the intervention group (*p* = 0.03), and there were race differences between the two groups as well (*p* = 0.02). Finally, wear time differed between control and intervention groups (*p* = 0.01). The two groups did not differ in MVPA.

Table 2 presents the sample size and percent of participants for each potential moderator category (center-level demographics, center practices, social and physical environment characteristics) by control and intervention groups. Chi-squared analyses revealed significant baseline differences between the control and intervention centers for several demographic, center practice, and social and physical environment characteristics (Table 2).

At the center level, the control group had a higher percentage of males (75.8% vs. 43.1% greater than 50% male), a lower percentage of black students (37.9% vs. 48.4 greater than 70% black), and fewer parents with a 2-year degree (43.2% vs. 59.6% with greater than 50% of parents with 2-year degree). Children in intervention compared to control centers attended more private/commercial schools (59.5% vs. 43.1%). A higher percentage of children in control centers had lower baseline exposures to “physical activity restricted as punishment” (72.6% vs. 23.1% never restrict physical activity) and “TV/video watching” (92.2% vs. 73.5% allow TV ≤ once per week) and had higher exposures to “free time play” (62.6% vs. 45.7% greater than 46 min/day), “staff participation in children’s play time” (53.2% vs. 36.7% often/always participate), and “off-site trips” (43.2% vs. 9.4% more than once per month).

A significantly higher percentage of children in control compared to intervention centers were in preschools with lower baseline ECERS-R scores, indicating lower center quality (44.2% vs 14.9% with scores less than 6) and lower social environment scores based on a subset of ECERS-R items (81.1% vs. 38.8 with scores less than 6.7). At baseline a higher percentage of students in the intervention group was enrolled in centers with smaller class sizes (76.6% vs. 54.7% less than 19), larger classroom space (47.3% vs. 25.3% greater than 690 square feet), and larger playgrounds (43.1% vs. 19.0% greater than 11,178 square feet). At baseline a higher percentage of control centers had greater availability of portable equipment (53.7% vs. 41.9%).

Table 2 also presents the results of the mixed model ANCOVA with MVPA as the dependent variable and the results of the group by potential moderator interaction. There were significant (*p* < 0.05) interactions between groups and one center practice, which was teacher-led PA for 30-min sessions; between groups and two social environment characteristics, which were visible PA support and social environment (ECERS-R); and between groups and the physical environment characteristic of playground size.

**Figure 1:**
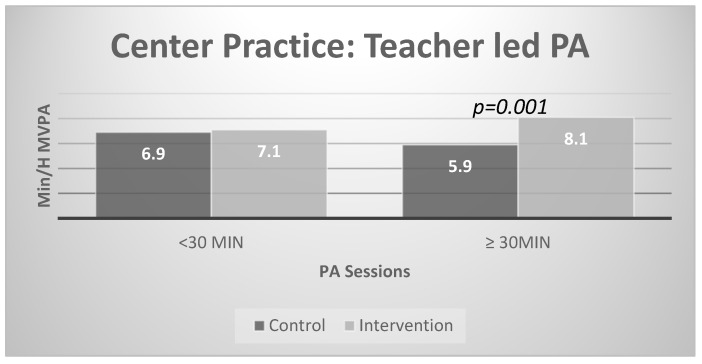
Interaction effects with teacher-led physical activity sessions.

Figure 1 presents the results of the stratified analysis for center practice. There were significant intervention effects for centers with high but not low baseline levels of teacher-led physical activity (≥ 30 min). Specifically, post-intervention physical activity was higher in the intervention group compared to the control in centers with more teacher-led PA at baseline (control = 5.9, intervention = 8.1).

**Figure 2:**
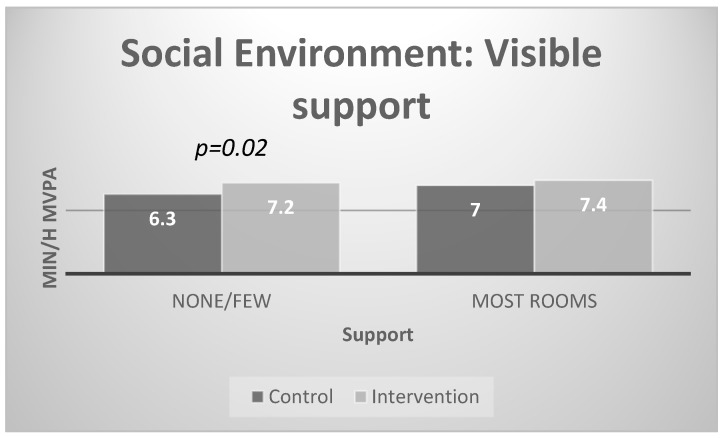
Interaction effects with visible support for physical activity.

**Figure 3:**
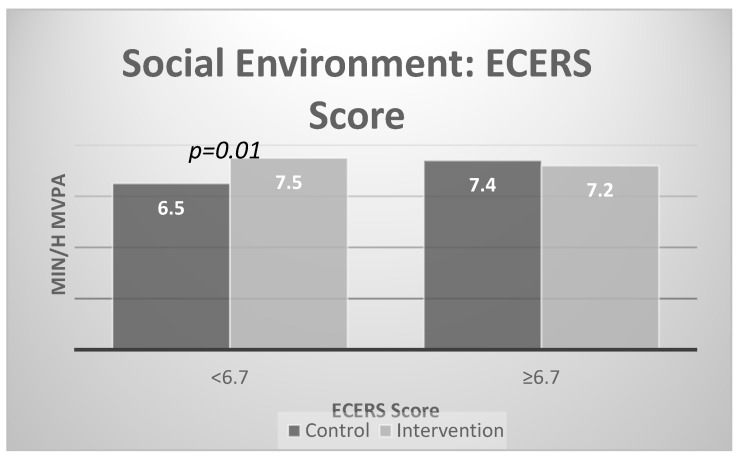
Interaction effects with social environment based on the Early Childhood Environment Rating Scale-Revised Edition (ECERS-R) scale subset.

Figure 2 and Figure 3 present the results of the stratified analyses for the social environment. There were significant intervention effects for centers with low levels of visible physical activity support (none, few) and low social environment (< 6.7). Specifically, post-intervention physical activity was higher in intervention compared to control centers with low social environment scores at baseline (7.5 min/hour vs. 6.5 min /hour of MVPA) and with low visible support at baseline (7.2 vs. 6.3 MVPA minutes). There were no differences in MVPA for children in control and intervention centers with high visible support and high social environment scores at baseline.

**Figure 4:**
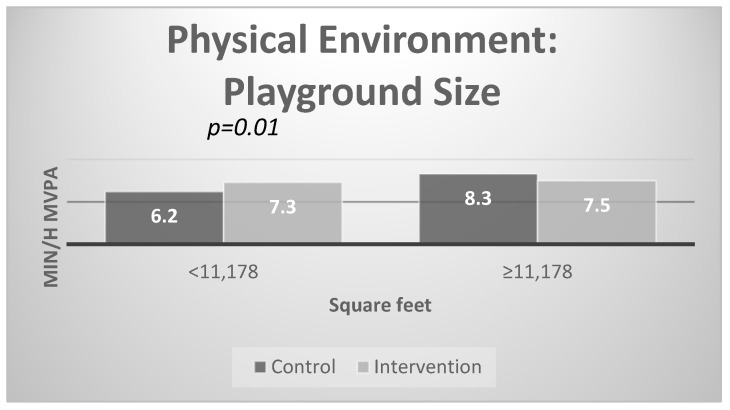
Interaction effects with physical environment: playground size.

Figure 4 presents the stratified results for the physical environment. There were significant intervention effects for centers with smaller playground size; specifically, at post-test, physical activity was higher in intervention compared to control centers with smaller playgrounds at baseline (7.3 vs. 6.2 min /hour of MVPA). There were no differences in MVPA for children in control and intervention centers with larger playgrounds at baseline.

## 4. Discussion

The novel finding of this study was that one center instructional practice, two social environment characteristics, and one physical environment characteristic at baseline moderated the effects of a successful physical activity intervention in center-based early childhood education and care (ECEC) programs. These findings are consistent with ecological [8,12] and systems [28,29] approaches which posit that contextual factors influence intervention implementation and provide additional support for the importance of taking the environmental context into account in early childhood program physical activity interventions [12,13,14,15,16,17]. They also underscore the need to examine baseline characteristics of the centers prior to undertaking an intervention and to conduct appropriate analyses to understand under what circumstances an intervention was effective [8,12,19,20]. This approach also allows interventionists to better fit interventions to contextual needs rather than apply pre-defined generic interventions.

*Center Instructional Practices.* The intervention was effective in centers with teachers who provided more physical activity opportunities at baseline. This result is consistent with previous work supporting the importance of teacher classroom behavior, especially teacher–child interaction [25].

SHAPES aimed to enhance teachers’ instructional practices to promote MVPA through integrating physical activity opportunities into the day. It is possible that teachers already involved in fostering physical activity with young children, even if less than optimally, made these modifications more easily, building on existing practices to promote physical activity. Hence, physical activity post-intervention was significantly higher in the intervention compared to control centers when both had high levels of teacher participation in physical activity at baseline. Policies to promote physical activity-promoting teacher instructional practices may “set the stage” for increased child physical activity.

*Center Social Environment.* The SHAPES intervention was more effective in centers with low baseline visible support for physical activity in the form of posters in classrooms and with lower baseline scores on the social environment items of the ECERS-R scale. The constructed ECERS-R social environment items we used included ratings such as greeting upon arrival and departure, encouraging children to communicate, staff–child interactions, and interactions among children. This moderated effect is consistent with findings on the importance of the social environment for early childhood educational and behavioral outcomes [25] and for promoting physical activity among young children [26]. It is also congruent with previous work showing that the social environment moderates intervention effects on educational outcomes [30] and an observational study showing interactions among physical activity intensity, child characteristics, social environment, and physical environments [8]. Results suggest that applying social environment interventions in centers with low ECERS-R scores may be beneficial.

The SHAPES intervention emphasized social support for physical activity, including messages promoting physical activity, teacher encouragement for physical activity, and teacher participation in physical activity. Increasing promotional messages in SHAPES was straightforward in centers that lacked them; this strategy may not be needed in centers that already have promotional messages.

*Center Physical Environment*. SHAPES was effective in centers with smaller playgrounds. This finding supports the importance of playground size in physical activity in young children, which is consistent with previous literature [26]. Given the importance of playground space for physical activity, we are not recommending the establishment of smaller playgrounds. Rather, SHAPES emphasized creative strategies to promote physical activity in the presence of space limitations, indoors and outdoors, and was specifically designed to address limitations in the physical environment.

*Variables without Moderating Influences.* In contrast to a review of previous studies [19] there were no moderating effects of child gender on SHAPES intervention effects; however, many of these studies were in older children and had methodological challenges. In this study, beyond gender, the following variables had no moderating effects on intervention outcomes: race, parent education, length of free play time, daily teacher-led physical activity, staff participation with children’s free play, restricting physical activity as punishment, TV use in the classroom, off-site trips, teacher physical activity training, overall center quality, number of children in the classroom, portable and fixed play equipment, classroom size, and public vs. private center designation. These results indicate that, in this study, variations in these characteristics at baseline did not influence the effects of the intervention, which is not to say that these characteristics are unimportant for this setting or for physical activity. It is difficult to compare these findings with previous results because there is limited literature on moderating factors in interventions [19], particularly in early childhood settings. Additional research in diverse childcare settings is needed to explore how these and other contextual factors moderate the effects of physical activity and other interventions. 

*Baseline Differences*. There were several baseline differences in factors that were assessed as potential moderators; however, few of these variables with baseline differences moderated intervention effects. The two that were found to moderate intervention effects, the revised ECERS-R social scale and playground size, were in a direction unfavorable to showing intervention effects in the intervention vs. control schools. The presence of numerous baseline differences among centers randomly assigned to intervention and control conditions underscores the complexity of this setting and the importance of considering contextual factors when intervening in these settings to promote physical activity in young children.

## 5. Strengths and Limitations

The centers were drawn from a limited geographic region within South Carolina, which may limit generalizability of the results presented here. However, this study is one of the first to examine a comprehensive set of moderating variables, including center demographics, baseline practices, and social and physical environmental characteristics. Results reported here also reflect center-level policies and practices reported by the director and may or may not reflect teacher-level classroom practices. Observational tools were developed for this project and lack assessment of psychometric properties. We used data-driven cut-points to dichotomize environmental variables which may have affected results and could potentially influence comparability with other studies. It is possible that variables not measured, such as the effects of weather, could also moderate effects of the intervention. Future studies should continue to examine organizational and environmental moderators on the effects of physical activity interventions in this setting, given the importance of environment for physical activity intensity in young children [8,12,13,14,15,16,17].

## 6. Conclusions

Baseline center-level instructional practices, social environment characteristics, and the physical environment moderated the effects of an effective physical activity intervention in center-based early childhood education and care (ECEC) programs. This is consistent with ecological and systems approaches and supports the importance of assessing contextual factors that may influence intervention outcomes [31]. Assessing baseline center-level practices and social and physical environment characteristics may aid efforts to match centers with evidence-based interventions likely to have a high yield in physical activity. An understanding of center practices and environmental characteristics may also suggest additional intervention strategies to be tested. Future research should explore strategies for assessing intervention contexts and examine combinations of intervention–context interactions [31,32] as a means for greater public health impact in early childhood education and care programs.

## Figures and Tables

**Table 1:** Baseline characteristics of 378 children: mean (SD) or percent. BMI: Body Mass Index; MVPA: moderate-to-vigorous physical activity.

Characteristic, Child	Control (*n* = 190)	Intervention (*n* = 188)	*p*-Value
Percent male	51.6%	48.9%	0.61
Age, years	4.5 (0.4)	4.5 (0.4)	0.56
BMI	16.3 (2.0)	16.3 (1.9)	0.92
Race			
White	39.0%	44.2%	0.02
Black	42.1%	46.8%	
Other	19.0%	9.0%	
Parent education, 2 or more years of college/tech school	54.7%	66.0%	0.03
Baseline PA			
MVPA, min/h	6.9 (2.8)	7.0 (2.7)	0.74
Wear time in center (hours)	5.5 (1.5)	5.1 (1.6)	0.01

**Table 2:** Sample size, frequencies of moderator variables by group and *p*-values from analysis of covariance (ANCOVA) models.

Moderator	Control (*n* = 190)	Intervention (*n* = 188)	Interaction: Group * Moderator *p*-Value
	*n* (%)	*n* (%)	
**Center Demographic Variables**			
Percent male			0.23
< 50%	**46 (24.2)**	**107 (56.9)**	
≥ 50%	**144 (75.8)**	**81 (43.1)**	
Percent black			0.25
< 70%	**118 (63.1)**	**97 (51.6)**	
≥ 70%	**72 (37.9)**	**91 (48.4)**	
Parent education			0.96
< 50% with a 2-year degree	**108 (56.8)**	**76 (40.4)**	
≥ 50% with a 2-year degree	**82 (43.2)**	**112 (59.6)**	
			
Public	**108 (56.8)**	**76 (40.4)**	0.96
Private school	**82 (43.1)**	**112 (59.5)**	
**Center Practice Variables**			
Free play time			0.18
< 46 min/day	**71 (37.4)**	**102 (54.3)**	
≥ 46 min/day	**119 (62.6)**	**86 (45.7)**	
Teacher-led PA			0.56
< Daily	99 (52.1)	88 (46.8)	
Daily	91 (47.9)	100 (53.2)	
Teacher-led PA			<0.001
< 30 min/session	134 (70.5)	136 (72.3)	
≥ 30 min/session	56 (29.5)	52 (27.7)	
Restrict PA as punishment			0.93
Sometimes	**52 (27.4)**	**130 (76.9)**	
Never	**138 (72.6)**	**39 (23.1)**	
TV use			0.99
> Once per week	**14 (7.8)**	**43 (26.5)**	
Once or less per week	**166 (92.2)**	**119 (73.5)**	
Staff participation with children’s free play			0.05
Rarely or sometimes	**89 (46.8)**	**107 (63.3)**	
Often/always	**101 (53.2)**	**62 (36.7)**	
Off-site trips			0.56
≤ One trip per month	**108 (56.8)**	**135 (90.6)**	
> One trip per month	**82 (43.2)**	**14 (9.4)**	
Teacher PA training			
Yes	104 (54.7)	98 (58.0)	0.92
No	86 (45.3)	71 (42.0)	
**Center Social Environment Variables**			
Visible support: posters			0.09
No or few	118 (62.1)	101 (62.4)	
In most rooms	72 (37.9)	61 (37.7)	
Quality			0.56
ECERS < 6	**84 (44.2)**	**28 (14.9)**	
ECERS ≥ 6	**106 (55.8)**	**160 (85.1)**	
ECERS social environment			
< 6.7	**154 (81.1)**	**73 (38.8)**	0.03
≥ 6.7	**36 (18.9)**	**115 (61.2)**	
Children per class			0.99
19+	**86 (45.3)**	**44 (23.4)**	
< 19	**104 (54.7)**	**144 (76.6)**	
**Center Physical Environment Variables**			
Portable play equipment			0.58
0 (coded 1 and 2)	**88 (46.3)**	**104 (58.1)**	
1 (coded 3 and 4)	**102 (53.7)**	**75 (41.9)**	
Fixed playground equipment (from inventory)			0.14
< 7	104 (54.7)	86 (45.7)	
≥ 7	86 (45.3)	102 (54.3)	
Playground size			0.02
< 11,178 square feet	**154 (81.0)**	**107 (56.9)**	
≥ 11,178 square feet	**36 (19.0)**	**81 (43.1)**	
Classroom size			0.64
< 690 square feet	**142 (74.7)**	**99 (52.7)**	
≥ 690 square feet	**48 (25.3)**	**89 (47.3)**	

Adjusting for wave, and random statement with center nested in group. **Boldface** indicates significant chi-squared between moderator categories.
